# GRASSP: RNA language model–enhanced graph attention with adaptive gating for RNA–small molecule binding site prediction

**DOI:** 10.1093/bioinformatics/btag638

**Published:** 2026-08-22

**Authors:** Thi Lan Nguyen, Nguyen Quoc Khanh Le

**Affiliations:** School of Electrical Engineering and Computer Science, The University of Queensland, St Lucia, Brisbane, QLD 4072, Australia; AIBioMed Lab, Taipei Medical University, Taipei 110, Taiwan; AIBioMed Lab, Taipei Medical University, Taipei 110, Taiwan; In-Service Master Program in Artificial Intelligence in Medicine, College of Medicine, Taipei Medical University, Taipei 110, Taiwan; Translational Imaging Research Center, Taipei Medical University Hospital, Taipei 110, Taiwan

## Abstract

**Motivation:**

RNA–small molecule binding site prediction is crucial for targeted drug discovery. Sequence-based methods are efficient but often fail to capture structural dependencies between nucleotides, whereas structure-aware graph models can better represent spatial interactions but typically rely on complex structural annotations and multi-stage preprocessing pipelines. We therefore developed GRASSP, a streamlined hybrid deep learning framework that integrates pretrained RNA language model (LM) representations with adaptive graph refinement.

**Results:**

GRASSP leverages nucleotide embeddings and predicted secondary-structure features from a pretrained RNA LM to construct spatial RNA graphs, followed by a lightweight two-step graph attention refinement module with adaptive gating to capture local and contextual nucleotide dependencies. Across four benchmark datasets (TE18, HARIBOSS, TL12, and JL10), GRASSP generally outperformed state-of-the-art baselines, with improvements of up to 24.1% in AUC and 44.5% in MCC. Ablation analyses showed that pretrained RNA representations provided the dominant predictive contribution, while spatial graph refinement offered complementary but dataset-dependent benefits. These results demonstrate that GRASSP provides a competitive framework for integrating pretrained RNA representations with spatial structural context while reducing reliance on additional handcrafted structural annotations.

**Availability:**

Code and datasets are publicly available at https://github.com/langiocn/GRASSP, with an archival snapshot available on Zenodo at https://doi.org/10.5281/zenodo.21888291.

## 1 Introduction

RNA-small molecule binding site prediction plays an important part in drug discovery, since RNA has been identified as a promising therapeutic target for a number of human diseases Esteller (2011). Despite the significant success of protein-targeted drug discovery, protein-coding genes account for only 1.5% of the human genome, and only 10%–15% of these proteins are considered druggable Cheng *et al*. (2007), Fan *et al*. (2023). In contrast, more than 95% of the genome is transcribed into RNA, which emphasizes its enormous potential for RNA-targeted drug development Chen *et al*. (2024), Jung *et al*. (2025). RNA-small molecule binding site prediction helps in the virtual screening of candidate molecules, enhances drug selectivity, and speeds up the drug development process Childs-Disney *et al*. (2022). However, experimental identification of RNA binding regions is often time-consuming, laborious, and expensive, making it challenging to explore on a large scale. Therefore, there is a need for efficient and accurate computational methods to predict RNA-small molecule binding sites. Several computational approaches have been developed for RNA–small molecule binding site prediction. RLBind Wang *et al*. (2023) integrates sequence and structural features using convolutional neural networks to capture both long- and short-range interactions. However, these methods struggle to capture complex nucleotide interactions under multi-view and multi-scale structural settings. To overcome this challenge, MVRBind Chen *et al*. (2025) uses a multi-view, multi-scale graph architecture that integrates primary, secondary, and tertiary structural information. Although this approach is effective, it involves external web-based secondary structure annotation (RNApdbee) and a multi-stage preprocessing procedure, which may increase implementation complexity. Conversely, more recent methods that are sequence-based utilize large RNA language models, such as ERNIE-RNA Yin *et al*. (2025), RNABERT Akiyama and Sakakibara (2022), and RiNALMo Penić *et al*. (2025) to learn contextualized embeddings of the sequence and use simple predictors, such as CoBRA Jang and Shin (2026). These methods are computationally efficient and do not rely on structural information, but they do not model structural relationships between nucleotides, which could be a drawback in modeling complex spatial dependencies. To address these limitations, we introduce GRASSP, a hybrid deep learning model that leverages the strong RNA sequence embeddings and the predicted secondary structure features obtained from a pre-trained language model, with a simple graph attention model to capture complex nucleotide relationships. By learning secondary features directly from the pretrained model and not requiring additional tertiary structural features, such as topological properties and solvent accessible surface areas (ASAs) as input node features, GRASSP helps to reduce the need for external structural annotation tools. This design simplifies preprocessing and feature extraction, while still enabling the model to capture complex interactions among RNA residues. We evaluate GRASSP against state-of-the-art methods on four benchmark datasets. Experimental results across four benchmark datasets demonstrate competitive or superior overall performance relative to existing methods, although the magnitude of improvement varies across datasets and evaluation metrics. Besides achieving superior prediction performance, GRASSP holds strong practical potential to accelerate RNA-targeted drug discovery by optimizing early screening workflows.

In summary, the main contributions of this work are as follows:

- We develop a hybrid deep learning framework for RNA–small molecule binding site prediction that integrates pretrained RNA representations with spatial graph-based modeling.
- We combine RNA language model embeddings with predicted secondary-structure-derived features, reducing reliance on additional handcrafted structural descriptors and external structural annotation procedures.
- We introduce a lightweight two-step graph attention refinement module with shared parameters, residual connections, and adaptive gating to integrate nucleotide representations across successive message-passing stages.
- Extensive evaluations across four benchmark datasets demonstrate competitive or superior overall performance compared with existing methods, although the magnitude of improvement varies across datasets and evaluation metrics.
- Comprehensive ablation and interpretability analyses characterize the contributions of different input modalities and model components, while also revealing dataset-dependent effects of spatial graph refinement.

## 2 Related works

### 2.1 RNA embeddings from a pretrained large model

Large pretrained RNA language models have been used in several recent studies to extract informative representations for the prediction of RNA-binding sites. For instance, RLsite Sun *et al*. (2025) uses ERNIE-RNA to obtain sequence embeddings, which are then fed into a multilayer perceptron (MLP) for prediction. Similarly, CoBRA Jang and Shin (2026) shows that competitive performance can be attained with a simplified architecture by using RiNALMo-derived Penić *et al*. (2025) sequence representations and a straightforward MLP classifier. However, these approaches directly use pretrained sequence embeddings and often overlook the explicit modeling of complex interactions among these features from multiple structural perspectives. In parallel, MVRBind integrates sequence information with various structural features within a multi-view, multi-scale graph convolutional framework. While this design achieves strong predictive performance, it introduces additional complexity in feature engineering and depends on multiple external software tools for structural annotation. To balance these trade-offs, GRASSP combines sequence embeddings with predicted secondary structural features derived directly from ERNIE-RNA, thereby reducing feature extraction effort while capturing complex interactions within a graph attention framework.

### 2.2 Graph-based models and adaptive feature integration

Graph-based approaches have been widely explored to model residue-level interactions in RNA structures. RNet Liu *et al*. (2024) introduced a distance-based dynamical graph algorithm to simulate node interactions derived from tertiary structural information. The extracted graph features were subsequently integrated into an ensemble learning framework for classification. RNABind Zhu *et al*. (2025) leverages large pretrained models to obtain RNA sequence embeddings and employs an Equivariant Graph Convolutional Layer (EGCL) to capture spatial relationships and geometric properties, followed by a prediction module to generate final binding outcomes. RLsite Sun *et al*. (2025) adopts a Graph Attention Network (GAT) to model secondary and tertiary structural features. Meanwhile, MVRBind Chen *et al*. (2025) employs a multi-layer GCN-based architecture to capture complex dependencies among residues from multiple perspectives, incorporating multi-scale relational edges (e.g. first-, second-, and third-order connections) alongside a transformer module and a dedicated self-attention fusion to enhance representation learning. Despite these advances, existing graph-based models typically aggregate multi-layer representations through direct concatenation or sequential propagation. To the best of our knowledge, no prior study has explored a two-layer weight-sharing GAT architecture combined with an adaptive gating mechanism for feature fusion, as implemented in GRASSP. By introducing a learnable gate to integrate representations from different propagation stages, this mechanism allows the model to learn the relative importance of representations from different propagation stages.

## 3 Proposed method

### 3.1 Overview

We formulate RNA–small molecule binding site prediction as a residue-level supervised learning problem. Given an RNA molecule with *N* nucleotides, the model takes as input (i) sequence embeddings $P\in R^{N\times d_{e}}$, (ii) predicted secondary structure–derived features $S\in R^{N\times d_{s}}$, and (iii) a graph edge set *E* encoding relationships between nucleotides. Here, $d_{e}$ and $d_{s}$ denote the dimensionalities of the sequence embedding and secondary structure feature spaces, respectively. The RNA molecule is represented as a graph G=(V,E), where each node corresponds to a nucleotide, and node features are obtained by concatenating the corresponding sequence and secondary structure representations. The objective is to learn a function $f_{\theta }(P,S,E)$ that outputs a binding probability for each nucleotide, and each nucleotide is assigned a binary label, where 1 indicates binding and 0 indicates non-binding, based on the predicted probability.

### 3.2 Dataset preparation

We conducted experiments on five publicly available RNA–small molecule binding site datasets: TR60, TE18, HARIBOSS, TL12, and JL10. Dataset statistics are summarized in Table 1. The TR60 and TE18 datasets are derived from the RNAsite Su *et al*. (2021) benchmark, which was constructed by collecting RNA–small molecule complex structures from the Protein Data Bank (PDB) Burley *et al*. (2019) and filtering for RNA chains interacting with small molecules while excluding water molecules Su *et al*. (2021). After removing very short or long RNA chains and those containing only crystallization additives, 712 RNA chains were retained. Redundancy was further reduced using TM-score–based structural clustering, resulting in 78 representative RNA structures Chen *et al*. (2025). Subsequently, these structures were divided into two subsets: the 60 RNAs comprised the TR60 dataset, while the remaining 18 RNAs comprised the TE18 dataset Su *et al*. (2021). We further included the HARIBOSS dataset Panei *et al*. (2022), which was split based on structural similarity following RNABind Zhu *et al*. (2025), with nucleotide label counts reported in Table 2. In addition, we used the TL12 and JL10 datasets from the ZeSTa study Gao *et al*. (2024). Following CoBRA Jang and Shin (2026), the JL10 was constructed from RNA–small molecule complex structures deposited after January 2021 and is characterized by junction-loop motifs with high structural complexity, whereas the TL12 consists of loop-free RNA structures with lower structural complexity. In this research, a nucleotide is labeled as a binding site residue if at least one of its atoms lies within 4 Å of any atom in the bound ligand, following MVRBind.

**Table 1:** Statistics of the datasets used in this study.

Dataset	RNA chains	Nucleotides	Binding	Non-binding
TR60	60	3054	950	2104
TE18	18	610	207	403
HARIBOSS	353	35 114	4958	30 156
JL10	10	821	264	557
TL12	12	515	183	332

Binding and non-binding denote nucleotide-level labels indicating whether a residue participates in small molecule binding.

**Table 2:** Nucleotide label counts for the HARIBOSS dataset splits.

Set	Train (+/–)	Validation (+/–)	Test (+/–)
Set1	3583:22330	565:4406	810:3420
Set2	4174:23132	350:2274	434:4750
Set3	3809:18735	376:4261	773:7160
Set4	3695:21902	675:1387	588:6867

Values are reported as binding: non-binding nucleotide counts.

### 3.3 Model architecture

**Figure 1:**
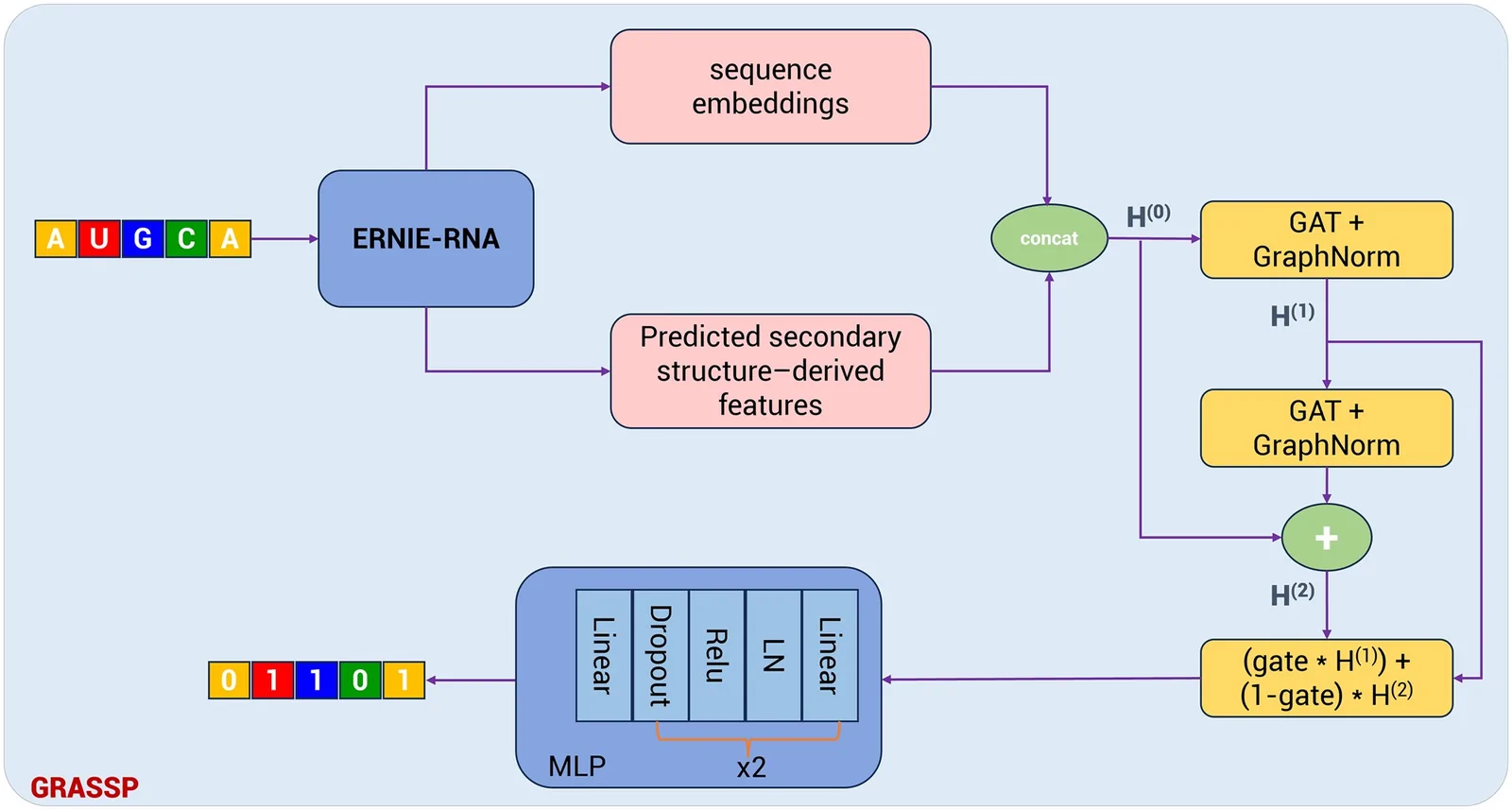
Overview of the proposed GRASSP architecture.

In this study, we propose *GRASSP* (Graph-based RNA Small Molecule Binding Site Prediction) for predicting small-molecule binding sites. The overall architecture of GRASSP is shown in Fig. 1. Specifically, GRASSP employs a two-layer GAT architecture in which a single GATConv layer is applied twice with shared parameters. This weight-sharing strategy reduces the number of trainable parameters, thereby improving generalization on relatively limited RNA–small molecule binding datasets. A two-layer message-passing scheme enables GRASSP to capture complementary local and contextual structural information for RNA–small molecule binding-site prediction while reducing the risk of over-smoothing associated with deeper graph networks. Furthermore, unlike existing methods that rely on conventional residual connections or static feature aggregation, GRASSP introduces an adaptive gating mechanism to dynamically integrate the representations obtained after the first and second graph propagation steps. By learning a node-specific gating vector from the concatenated representations ($H^{\left(1\right)}$ and $H^{\left(2\right)}$), the model adaptively balances immediate neighborhood information with broader structural context before residue-level binding-site prediction.

Compared with RLSite, GRASSP provides a more integrated use of RNA language model representations for RNA–small molecule binding-site prediction. Unlike RLSite, which additionally utilizes solvent-accessible surface area (ASA) and torsion (TOR) features, GRASSP does not require these additional structural features. RLSite primarily uses GAT to encode structural features and only incorporates RNA-ERNIE sequence embeddings at a later prediction stage. In contrast, GRASSP derives both sequential and predicted secondary-structure features from RNA-ERNIE and fuses them before graph attention. This early fusion enriches each nucleotide node with sequence–structure information, enabling GAT to learn nucleotide interactions and attention weights from a joint representation rather than from structural features alone. While RLSite mainly relies on a static feature aggregation followed by MLP-based prediction, GRASSP applies adaptive gating fusion to combine the representations produced by two successive GAT updates, allowing the model to dynamically integrate different graph-based views of each nucleotide before binding-site classification.

### 3.4 Input representation

Given an input RNA sequence $R=\{e_{1},e_{2},\ldots ,e_{N}\}$ with *N* nucleotides, we first extract nucleotide-level features using a pretrained RNA language model. ERNIE-RNA Yin *et al*. (2025) is used to obtain a contextual sequence embedding $p_{i}\in R^{d_{\text{rna}}}$ and secondary–structure derived features $s_{i}\in R^{d_{\text{ss}}}$ for each nucleotide, where $d_{\text{rna}}=768$ and $d_{\text{ss}}=4$. Based on these features, each RNA is represented as a graph G=(V,E) with *N* nodes, where each node corresponds to a nucleotide and is associated with the feature pair $\left(p_{i},s_{i}\right)$ and a node-level binding label (1 or 0). Graph edges are constructed according to spatial proximity: three-dimensional nucleotide coordinates obtained from structure files are used to connect each node to its *k* nearest neighbors in Euclidean space. Following the previous study Chen *et al*. (2025), *k* = 8 achieved the best performance. This graph representation captures both contextualized sequence features and secondary-structure context, as well as their spatial relationships.

### 3.5 Feature fusion

Sequence features and secondary structural features are then concatenated and projected into a shared hidden space of dimension *H* to obtain the initial representation $H^{\left(0\right)}$, enriching node embeddings.

### 3.6 Graph representation learning

We use graph attention networks (GAT) Veličković *et al*. (2017) for message passing, as they assign different weights to neighboring nodes rather than treating all neighbors equally, which helps capture the varying importance of node interactions. For a node *i*, a single GAT layer computes


(1) $$h_{i}^{\prime }=\sum_{j\in N(i)}\alpha_{ij}\;Wh_{j},\;\alpha_{ij}=\frac{\;\text{exp}(e_{ij})}{\sum_{k\in N(i)}\;\text{exp}\;(e_{ik})},$$

where N(i) denotes the neighborhood of *i*, W is a learnable linear map, and $e_{ij}$ is a learnable attention score computed from node features. In our model, we apply two successive GAT blocks:


(2) $$H^{\left(1\right)}=\phi (\text{GN}(\text{GAT}(H^{\left(0\right)},E),\mathrm{batch})),$$

(3) $$H^{\left(2\right)}=\phi (\text{GN}(\text{GAT}(H^{\left(1\right)},E),\mathrm{batch}))+H^{\left(0\right)},$$

where GN(·) denotes graph-wise normalization (GraphNorm) applied per graph in the mini-batch to normalize node features within each graph and improve training stability. The residual connection in Equation (3) facilitates gradient flow and helps preserve the initial node representations.

### 3.7 Gated fusion module

To combine information from the first and second GAT blocks, we concatenate $H^{\left(1\right)}$ and $H^{\left(2\right)}$ and compute a gate:


(4) $$Z=[H^{\left(1\right)}\|H^{\left(2\right)}]\in R^{N\times 2H},$$

(5) $$G=\sigma (ZW_{g}+b_{g})\in R^{N\times H},$$

(6) $$H=G⊙H^{\left(1\right)}+(1-G)⊙H^{\left(2\right)}\in R^{N\times H},$$

where σ(·) is the sigmoid function and ⊙ is element-wise multiplication. The gated mechanism adaptively determines the relative importance of representations from the two different GAT layers, enabling flexible modeling of local and contextual dependencies in RNA-binding site prediction.

### 3.8 Node-level binding site prediction

We use a two-stage MLP with LayerNorm, ReLU, and dropout to predict a binding logit for each nucleotide:


(7) $$o=f_{\text{head}}(H)\in R^{N},$$

where $o_{i}$ is the logit for nucleotide *i*. Binding probabilities for each nucleotide are obtained via $p_{i}=\sigma (o_{i})$.

### 3.9 Loss function

The model was trained end-to-end using a weighted binary cross-entropy loss to address class imbalance between binding and non-binding nucleotides. Given the predicted binding probability $p_{i}$ and the ground-truth label $y_{i}\in \{0,1\}$ for nucleotide *i*, the loss is defined as:


(8) $$L_{\text{WBCE}}=-\frac{1}{B}\sum_{i=1}^{B}\left[w_{+}y_{i}\;\text{log}(p_{i})+(1-y_{i})\;\text{log}(1-p_{i})\right],$$

where $w_{+}$ is the positive-class weight estimated from the training data, and *B* is the number of nucleotides in a training batch. All model parameters were optimized by minimizing $L_{\text{WBCE}}$ over the training set.

## 4 Experiments

### 4.1 Experimental setup

#### 4.1.1 Implementation details

All models were trained in a CPU-only environment. The official test sets were kept completely independent from model development to prevent data leakage. GRASSP was evaluated under three independent experimental settings, each corresponding to a different benchmark and following its respective evaluation protocol. (1) For the TE18 benchmark, GRASSP was trained on the TR60 dataset, with 90% of the data used for training and the remaining 10% for validation, and evaluated on the independent TE18 test set following the experimental setting of MVRBind. (2) For the HARIBOSS benchmark, each subset (SET1–SET4) was trained and evaluated using its official structure-based train, validation, and test splits. (3) For the TL12 and JL10 benchmarks, GRASSP was trained using the official training and validation sets constructed from the combined HARIBOSS and TR60 datasets following CoBRA. The model was then evaluated on the independent TL12 and JL10 test sets. Any overlapping sequences between the training and test sets were removed following the same preprocessing procedure as CoBRA. The model was optimized using AdamW with a cosine annealing warm restart scheduler. Binary cross-entropy with logits was employed as the loss function, where class imbalance was handled by assigning a positive class weight computed from the training data. To ensure a fair and rigorous comparison, all evaluations strictly utilized the exact same training and testing sets under the original protocols of the respective baselines. Consequently, all baseline results were directly adopted from the comparison tables reported in the corresponding previous studies.

#### 4.1.2 Hyperparameters

The learning rate was searched over {1e–3, 1e–4}, batch size over {1, 10}, hidden dimension over {64, 86, 128}, dropout over {0.3, 0.6}, and epochs were set to 100. This hyperparameter search space was identical across all benchmark datasets. For each dataset, the optimal parameters were selected independently based exclusively on the validation set.

#### 4.1.3 Model size and downstream training cost

The downstream GRASSP prediction network contains 68 801–174 465 trainable parameters, depending on the hidden dimension selected for each benchmark. In our CPU-only environment, downstream model training required approximately 2.5 minutes per run. This runtime excludes ERNIE-RNA feature extraction, data preprocessing, and spatial graph construction and therefore should not be interpreted as the end-to-end computational cost of the complete pipeline. We do not perform a systematic computational-efficiency comparison with competing methods.

#### 4.1.4 Evaluation metrics

As in previous work, we evaluate the model by Area Under the ROC Curve (AUC), Precision, Recall, and MCC on TE18, HARIBOSS (SET 1–SET 4), TL12, and JL10 datasets. MCC provides a balanced measure of binary classification performance, especially in the presence of class imbalance.

### 4.2 Performance on TE18

We compared GRASSP with six representative methods, including RNAsite Su *et al*. (2021), RBind Wang *et al*. (2018), RLBind Wang *et al*. (2023), Rnet Liu *et al*. (2024), RNABind Zhu *et al*. (2025), and MVRBind Chen *et al*. (2025). Following the same experimental protocol as MVRBind, GRASSP was trained on TR60 and tested on TE18. The dataset statistics in our study match those reported in MVRBind exactly, and the baseline results were directly adopted from their paper under the same setting. The performance comparison on the TE18 dataset is summarized in Table 3. Out of all the competing methods, GRASSP performs the best overall across the four evaluation metrics. GRASSP improves by 2.6% in precision, 24.3% in recall, and 2.3% in MCC when compared to the best baseline, MVRBIND. The notable increase in recall suggests that GRASSP is able to detect a much higher number of true binding residues. Simultaneously, under class-imbalanced conditions, the increase in MCC indicates a better balance between positive and negative predictions.

**Table 3:** Performance comparison on the TE18 dataset.

Model	TE18			
	AUC	Precision	Recall	MCC
RNAsite	0.710	0.550	0.159	0.147
Rbind	0.554	0.611	0.159	0.179
RLBind	0.719	0.632	0.320	0.277
Rnet	0.709	0.616	0.333	0.277
RNABind	0.717	0.463	0.341	0.263
MVRBind	0.745	0.645	0.342	0.351
**GRASSP**	**0.746**	**0.662**	**0.425**	**0.359**

Values in bold indicate the best-performing result for each evaluation metric.

### 4.3 Performance on HARIBOSS

To further evaluate GRASSP, we compare it with RLBind, RNet, RNABind, and MVRBind on SET1, SET2, SET3, and SET4 of the HARIBOSS dataset. Table 4 reports the performance across the four subsets. The results of these baseline methods are taken directly from the MVRBind study under the same training and testing settings. For GRASSP, we report the average performance over five runs with different random seeds. On all subsets and evaluation metrics, GRASSP outperforms MVRBind, the strongest baseline. Across SET1–SET4, GRASSP improves AUC by 3.9%–24.1%. Recall improves by 6.7%–74.4%, while precision gains range from 17.3% to 37.9%. Notably, MCC exhibits notable gains ranging from 16.5% to 44.5%, underscoring GRASSP’s advantage in handling class-imbalanced RNA–small molecule binding site prediction tasks. These consistent gains across all subsets show the effectiveness of combining contextualized sequence and structural embeddings within an efficient GAT architecture, as well as GRASSP’s generalization ability on the HARIBOSS benchmark.

**Table 4:** Performance comparison on the HARIBOSS dataset.

Model	HARIBOSS															
	SET1				SET2				SET3				SET4			
	AUC	Precision	Recall	MCC	AUC	Precision	Recall	MCC	AUC	Precision	Recall	MCC	AUC	Precision	Recall	MCC
RLBind	0.712	0.302	0.260	0.125	0.711	0.278	0.140	0.147	0.661	0.178	0.060	0.050	0.601	0.259	0.280	0.204
Rnet	0.731	0.364	0.295	0.188	0.717	0.178	0.034	0.044	0.673	0.204	0.185	0.112	0.657	0.212	0.047	0.066
RNABind	0.757	0.300	0.290	0.232	0.707	0.266	0.108	0.055	0.688	0.230	0.113	0.032	0.685	0.232	0.216	0.144
MVRBind	0.785	0.411	0.308	0.306	0.740	0.330	0.202	0.207	0.697	0.266	0.270	0.188	0.693	0.272	0.341	0.220
**GRASSP**	**0.816**	**0.541**	**0.537**	**0.424**	**0.776**	**0.387**	**0.278**	**0.253**	**0.781**	**0.323**	**0.288**	**0.219**	**0.860**	**0.375**	**0.390**	**0.318**

Values in bold indicate the best-performing result for each evaluation metric.

### 4.4 Performance on TL12 and JL10

To the best of our knowledge, CoBRA Jang and Shin (2026) is the most recent method (2026) for compound binding site prediction leveraging pretrained model. Therefore, we further evaluate GRASSP against ZeSTa Gao *et al*. (2024) and CoBRA Jang and Shin (2026) to provide a more comprehensive comparison. For a fair evaluation, we adopt the same experimental setup as reported in the CoBRA study. Specifically, the model is trained on the combined HARIBOSS and TR60 training sets, and evaluated on the JL10 and TL12 test sets. The results of ZeSTa and CoBRA are directly taken from the CoBRA paper. For GRASSP, results are averaged over five independent runs with different random seeds.

Table 5 presents the performance comparison between GRASSP and the recent state-of-the-art methods, ZeSTa and CoBRA, on the TL12 and JL10 test sets. GRASSP achieves the highest AUC, Precision, and MCC among the compared methods on both datasets, and the highest Recall on TL12. On JL10, however, CoBRA achieves a higher Recall than GRASSP (0.531 vs. 0.486). Compared with CoBRA, GRASSP improves AUC by 7.1% and 2.8% and MCC by 11.9% and 17.1% on TL12 and JL10, respectively. Overall, these results demonstrate competitive performance across the two independent test sets, with consistent improvements in AUC and MCC, while also indicating that the performance advantage does not extend uniformly to every evaluation metric.

**Table 5:** Performance comparison on the TL12 and JL10 datasets.

Model	TL12				JL10			
	AUC	Prec.	Rec.	MCC	AUC	Prec.	Rec.	MCC
ZeSTa	0.704	0.740	0.514	0.440	0.592	0.549	0.296	0.211
CoBRA	0.816	0.816	0.599	0.546	0.739	0.582	**0.531**	0.293
**GRASSP**	**0.874**	**0.827**	**0.646**	**0.611**	**0.760**	**0.587**	0.486	**0.343**

Values in bold indicate the best-performing result for each evaluation metric.

### 4.5 Ablation study

To assess the effectiveness of each component in the GRASSP framework, we perform ablation studies on the TE18, TL12, JL10, and HARIBOSS (SET2) datasets, as shown in Table 6.

**Table 6:** Ablation study on TE18, TL12, JL10, and HARIBOSS (SET2).

Model	TE18				TL12				JL10				HARIBOSS (SET2)			
	AUC	Prec.	Rec.	MCC	AUC	Prec.	Rec.	MCC	AUC	Prec.	Rec.	MCC	AUC	Prec.	Rec.	MCC
w/o seq_emb	0.626	0.468	0.314	0.147	0.756	0.692	0.323	0.314	0.621	0.476	0.299	0.167	0.604	0.214	0.151	0.103
w/o ss_emb	0.724	0.632	0.415	0.332	0.865	0.823	0.627	0.596	0.755	0.572	0.501	0.337	0.764	0.238	0.210	0.174
w/o gate (1 GAT)	0.720	0.608	0.367	0.288	0.835	0.765	0.616	0.541	0.735	0.500	0.499	0.312	0.724	0.341	0.132	0.103
w/o gate	0.738	0.640	0.387	0.322	0.864	0.825	0.603	0.581	0.754	0.576	0.476	0.327	0.767	0.314	0.273	0.193
w/o GAT (MLP only)	0.690	0.518	0.411	0.229	0.874	0.816	**0.654**	0.608	**0.773**	0.570	**0.555**	**0.359**	0.738	0.326	0.249	0.204
**GRASSP**	**0.746**	**0.662**	**0.425**	**0.359**	**0.874**	**0.827**	0.646	**0.611**	0.760	**0.587**	0.486	0.343	**0.776**	**0.387**	**0.278**	**0.253**

Values in bold indicate the best-performing result for each evaluation metric.

The removal of sequence embeddings (w/o seq_emb) results in the largest performance degradation. In terms of MCC, this results in a 59.1% decrease on TE18, 48.6% on TL12, 51.3% on JL10, and 59.3% on HARIBOSS (SET2), with a similar reduction in AUC values. This clearly indicates the importance of contextualized sequence embeddings.

Removing secondary structural features (w/o ss_emb) also results in a performance decrease, with MCC values decreasing by 7.5%, 2.45%, 1.75%, and 31.2% on the four datasets, respectively, confirming that structural features are complementary to sequence embeddings.

Without the gating mechanism and the simplification of the architecture to a single GAT layer (w/o gate (1 GAT)), there is a significant decrease in both AUC and MCC values (e.g. a 19.8% decrease in MCC on TE18 and a 11.5% decrease on TL12), clearly showing that the deeper graph modeling and adaptive gating mechanism improve the learning of structural features by adaptively combining the shallow and deep graph features.

Completely removing the gating mechanism and replacing it with a simple static concatenation causes a substantial performance degradation across all four benchmark datasets compared with the full GRASSP model, as shown in Table 6. In specific, the MCC decreases by 10.3%, 4.9%, 4.67%, and 23.7% on TE18, TL12, JL10, and HARIBOSS (SET2), respectively. Similar declines are observed for AUC, Precision, and Recall after removing the gating mechanism. These results Illustrate the effectiveness of the proposed adaptive gating mechanism within the GRASSP architecture. Rather than statically concatenating feature representations, the gating mechanism dynamically selects and integrates complementary information from different feature views, which may allow the model to focus on the most informative features for RNA–small molecule binding-site prediction.

**Figure 2:**
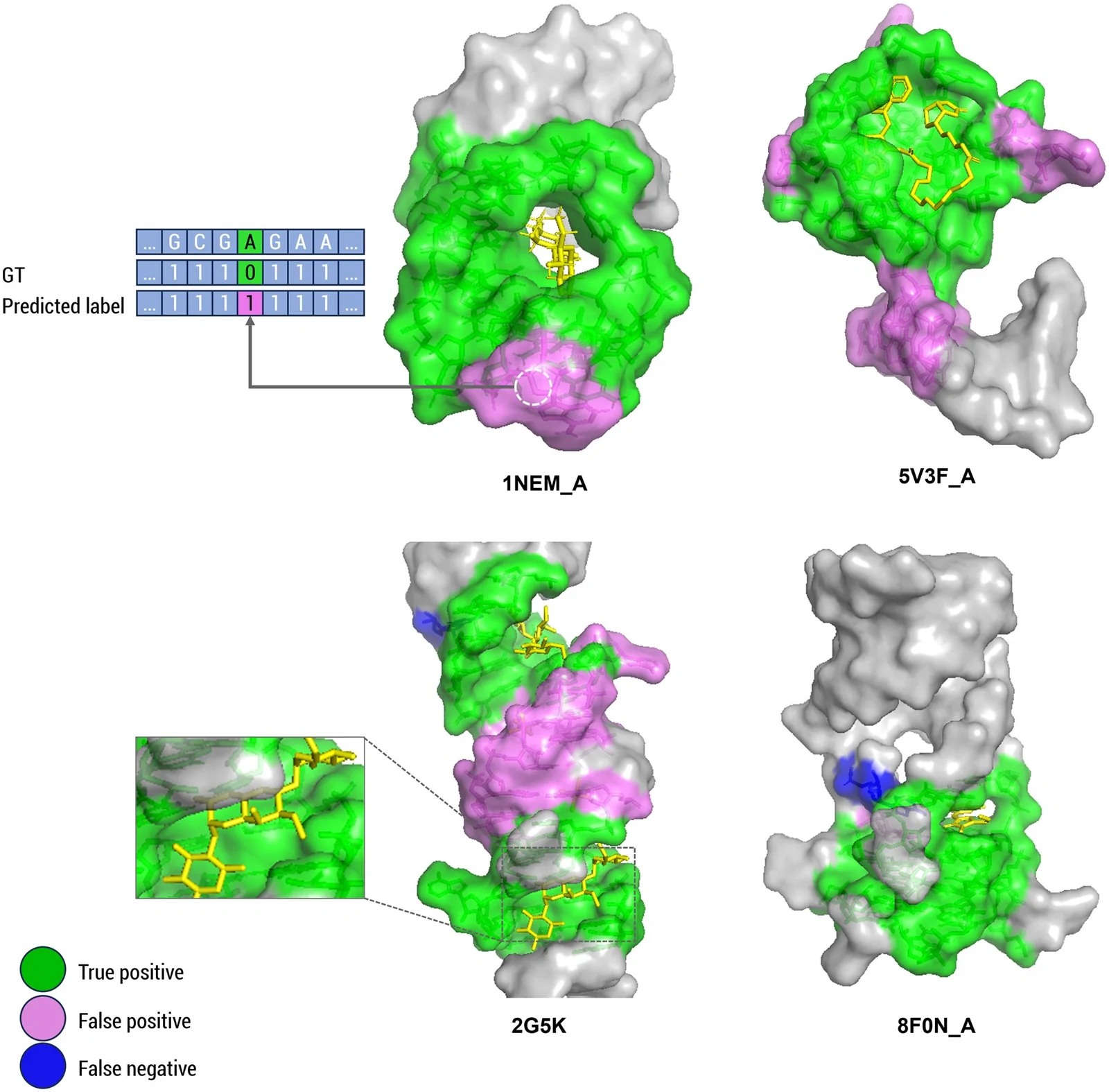
Structural visualization of GRASSP predictions using PyMOL. Ligands are shown in yellow, while true positives are in green, false positives in violet, and false negatives in blue.

Notably, the MLP-only variant (w/o GAT (MLP only)) even slightly improves over the full model on JL10. We speculate that this is due to some dataset-specific properties. Specifically, JL10 is the smallest dataset, containing only 10 RNAs. It is characterized by complex junction loop structures that exhibit high structural complexity Jang and Shin (2026), which may cause standard 3D coordinate-based graphs to capture more topological noise than useful signal. In these highly curved loop regions, constructing graphs based on a fixed number of spatial neighbors (k = 8) may inadvertently link structurally distant or functionally irrelevant residues. Consequently, graph-based message passing could add less value or even increase the noise inherent in the graph structure, making it easier for a simpler model to generalize slightly better. However, this is not the case for the other datasets, especially TE18 and HARIBOSS (SET2), where pocket residues are heavily clustered and exhibit clean spatial connectivity, as exemplified by 1NEM_A and 5V3F_A in Fig. 2. In these data-rich or topologically dense scenarios, removing graph modeling leads to a significant performance drop (e.g. −36.2% on TE18 and −19.4% on HARIBOSS for MCC).

In summary, although there is a mild exception for JL10, the full GRASSP model performs better and more robustly on all datasets, suggesting that the combination of sequence embeddings, structural information, multi-layer GAT, and gating is beneficial for predictive performance.

### 4.6 Case study

To better examine the interpretability of GRASSP, we plot four example RNA-ligand complexes (1NEM_A, 5V3F_A, 2G5K, and 8F0N_A, “_A” indicates chain A) from the TE18, HARIBOSS, and TL12 test sets using the PyMOL molecular visualization tool Schrodinger (2015), as shown in Fig. 2. In these plots, the ligands are colored yellow, and the true positives (binding residues), false positives, and false negatives are colored green, violet, and blue, respectively. These examples were selected because their prediction metrics closely align with the model’s average performance across the respective datasets, as well as capture both successful identifications and typical error modes (false positives and false negatives).

In all four examples, the predicted binding residues overlap well with the ligand-binding areas. Specifically, for 1NEM_A and 5V3F_A, GRASSP is able to predict most of the binding residues around the ligands, which together define spatially consistent binding pockets. Similar observations can be made for 2G5K and 8F0N_A, where the predicted binding residues are also concentrated around the ligand interfaces. These results indicate that the model effectively captures the structural context of RNA-small molecule interactions and localizes binding sites with high spatial consistency.

### 4.7 False positive analysis on 1NEM_A (nucleotide 11)

In 1NEM_A, we find a typical false positive at nucleotide 11 (violet in Fig. 2). Further analysis reveals that this false positive may not be attributed to the aggregation of the graph structure, as removing the neighborhood information has only a slight impact on the prediction confidence (0.7041 vs. 0.6990). Rather, the prediction is dominantly affected by the RNA embedding of the nucleotide itself.

Modality ablation also shows that setting the RNA embedding to zero decreases the predicted probability (0.7041–0.6361), while the effect of setting the secondary structure embedding to zero is minimal. In the RNA embedding space, nucleotide 11 is more similar to the centroid of binding residues than to non-binding residues, and most of its similar residues are true binding nucleotides. This phenomenon might suggest that the nucleotide is located in a region of feature space that overlaps with binding-like features.

Based on the above findings, the false positive might be caused by the similarity of features between binding and non-binding nucleotides, rather than the mislocalization of structural information. In future work, we could attempt to achieve a more balanced fusion of RNA and secondary structure information, add more structural or physicochemical features, and examine training approaches that deal with these negative examples more effectively.

## 5 Conclusion

In this study, we introduced GRASSP, a deep learning framework for RNA-small molecule binding site prediction that fuses sequence embeddings and predicted secondary-structure features extracted from the pretrained ERNIE-RNA model with a two-step graph attention refinement network equipped with an adaptive gating mechanism. By leveraging ERNIE-RNA to provide both contextual sequence representations and structural cues, GRASSP helps to reduce efforts in feature engineering. Extensive experiments on four benchmark datasets show that GRASSP consistently achieves better performance compared with representative existing methods across all evaluation metrics. The case study further demonstrates the model’s ability to correctly localize ligand-binding regions and provides insight through the analysis of false positive predictions.

While these findings demonstrate the effectiveness of GRASSP, certain limitations still need to be acknowledged. Notably, in addition to sequence features, GRASSP also relies on predicted secondary structure features extracted from ERNIE-RNA. This means the robustness of the model could be potentially affected when such features are imperfect or noisy. Future work will further investigate this dependency to assess the framework’s resilience when structural predictions carry errors, while also focusing on the improvement of structural input quality. Along with this, we aim to explore more automated and simplified strategies to further enrich residue-level representations. Integrating alternative large RNA language models within the proposed framework may also enhance performance. In addition, evaluating GRASSP on broader benchmark collections would provide further validation once they become publicly available. Furthermore, the performance of our two-layer GAT with adaptive gating remains limited on the JL10 dataset. Future work will explore various configurations of spatial neighbors (*k*) to identify more effective ways to handle topologically complex graphs like JL10.

## Author contributions

Thi Lan Nguyen (Conceptualization [Equal], Data curation [Equal], Formal analysis [Equal], Investigation [Lead], Methodology [Lead], Software [Lead], Visualization [Lead], Writing—original draft [Lead], Writing—review & editing [Equal]), and Nguyen Quoc Khanh Le (Conceptualization [Equal], Data curation [Equal], Funding acquisition [Lead], Project administration [Lead], Resources [Equal], Supervision [Lead], Writing—review & editing [Equal])

## Data Availability

Code and datasets are publicly available at https://github.com/langiocn/GRASSP, with an archival snapshot available on Zenodo at https://doi.org/10.5281/zenodo.21888291. The repository provides the implementation and scripts used in our experiments.
